# Teaching the science of learning

**DOI:** 10.1186/s41235-017-0087-y

**Published:** 2018-01-24

**Authors:** Yana Weinstein, Christopher R. Madan, Megan A. Sumeracki

**Affiliations:** 1 Department of Psychology, University of Massachusetts Lowell, Lowell, MA USA; 2 Department of Psychology, Boston College, Chestnut Hill, MA USA; 3 School of Psychology, University of Nottingham, Nottingham, UK; 4 Department of Psychology, Rhode Island College, Providence, RI USA

**Keywords:** Education, Learning, Memory, Teaching

## Abstract

The science of learning has made a considerable contribution to our understanding of effective teaching and learning strategies. However, few instructors outside of the field are privy to this research. In this tutorial review, we focus on six specific cognitive strategies that have received robust support from decades of research: spaced practice, interleaving, retrieval practice, elaboration, concrete examples, and dual coding. We describe the basic research behind each strategy and relevant applied research, present examples of existing and suggested implementation, and make recommendations for further research that would broaden the reach of these strategies.

## Significance

Education does not currently adhere to the medical model of evidence-based practice (Roediger, 2013). However, over the past few decades, our field has made significant advances in applying cognitive processes to education. From this work, specific recommendations can be made for students to maximize their learning efficiency (Dunlosky, Rawson, Marsh, Nathan, & Willingham, 2013; Roediger, Finn, & Weinstein, 2012). In particular, a review published 10 years ago identified a limited number of study techniques that have received solid evidence from multiple replications testing their effectiveness in and out of the classroom (Pashler et al., 2007). A recent textbook analysis (Pomerance, Greenberg, & Walsh, 2016) took the six key learning strategies from this report by Pashler and colleagues, and found that very few teacher-training textbooks cover any of these six principles – and none cover them all, suggesting that these strategies are not systematically making their way into the classroom. This is the case in spite of multiple recent academic (e.g., Dunlosky et al., 2013) and general audience (e.g., Dunlosky, 2013) publications about these strategies. In this tutorial review, we present the basic science behind each of these six key principles, along with more recent research on their effectiveness in live classrooms, and suggest ideas for pedagogical implementation. The target audience of this review is (a) educators who might be interested in integrating the strategies into their teaching practice, (b) science of learning researchers who are looking for open questions to help determine future research priorities, and (c) researchers in other subfields who are interested in the ways that principles from cognitive psychology have been applied to education.

While the typical teacher may not be exposed to this research during teacher training, a small cohort of teachers intensely interested in cognitive psychology has recently emerged. These teachers are mainly based in the UK, and, anecdotally (e.g., Dennis (2016), personal communication), appear to have taken an interest in the science of learning after reading *Make it Stick* (Brown, Roediger, & McDaniel, 2014; see Clark (2016) for an enthusiastic review of this book on a teacher’s blog, and “Learning Scientists” (2016c) for a collection). In addition, a grassroots teacher movement has led to the creation of “researchED” – a series of conferences on evidence-based education (researchED, 2013). The teachers who form part of this network frequently discuss cognitive psychology techniques and their applications to education on social media (mainly Twitter; e.g., Fordham, 2016; Penfound, 2016) and on their blogs, such as Evidence Into Practice (https://evidenceintopractice.wordpress.com/), My Learning Journey (http://reflectionsofmyteaching.blogspot.com/), and The Effortful Educator (https://theeffortfuleducator.com/). In general, the teachers who write about these issues pay careful attention to the relevant literature, often citing some of the work described in this review.

**Fig. 1:**
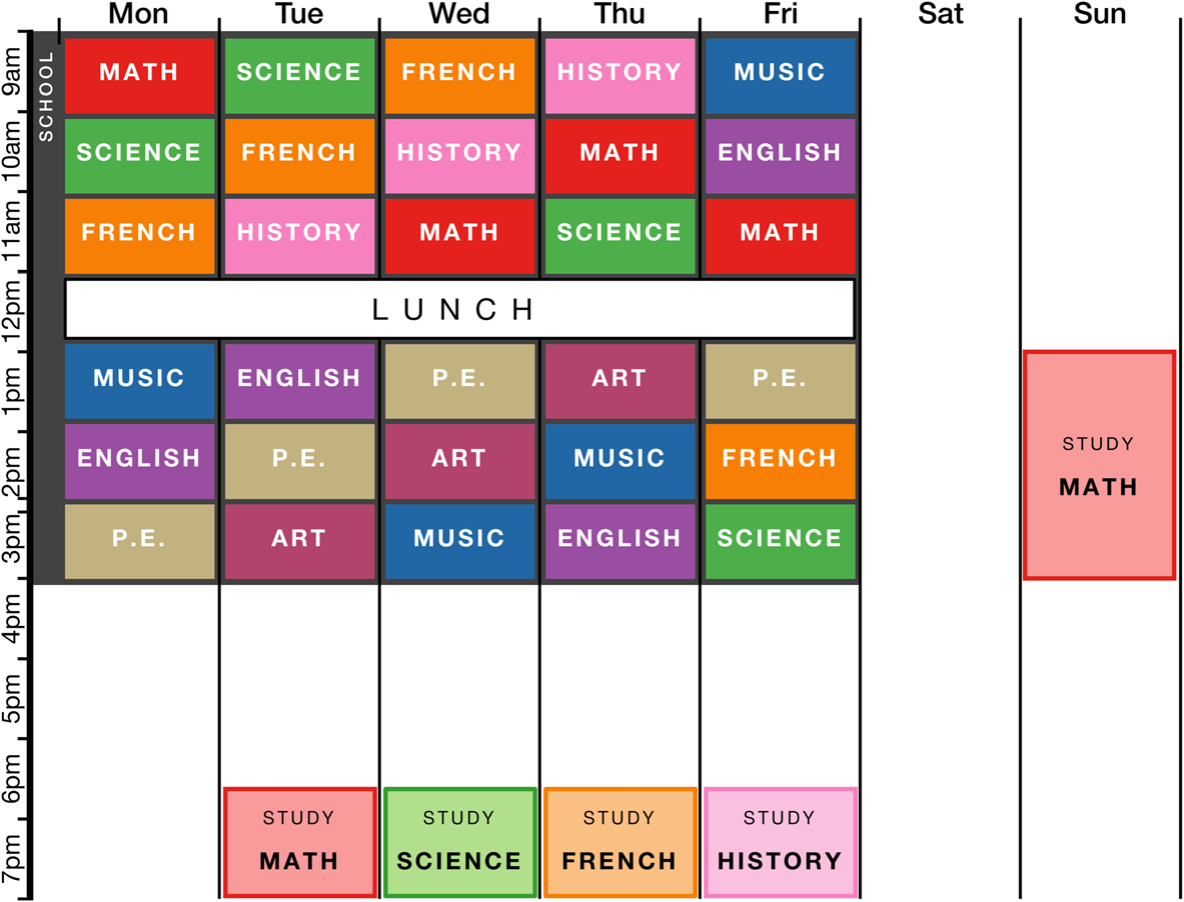
Spaced practice schedule for one week. This schedule is designed to represent a typical timetable of a high-school student. The schedule includes four one-hour study sessions, one longer study session on the weekend, and one rest day. Notice that each subject is studied one day after it is covered in school, to create spacing between classes and study sessions. *Copyright note:* this image was produced by the authors

**Fig. 2:**
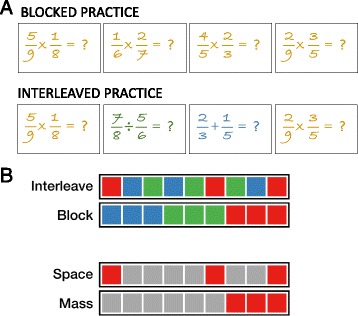
**a** Blocked practice and interleaved practice with fraction problems. In the blocked version, students answer four multiplication problems consecutively. In the interleaved version, students answer a multiplication problem followed by a division problem and then an addition problem, before returning to multiplication. For an experiment with a similar setup, see Patel et al. (2016). *Copyright note:* this image was produced by the authors. **b** Illustration of interleaving and spacing. Each color represents a different homework topic. Interleaving involves alternating between topics, rather than blocking. Spacing involves distributing practice over time, rather than massing. Interleaving inherently involves spacing as other tasks naturally “fill” the spaces between interleaved sessions. *Copyright note:* this image was produced by the authors, adapted from Rohrer (2012)

**Fig. 3:**
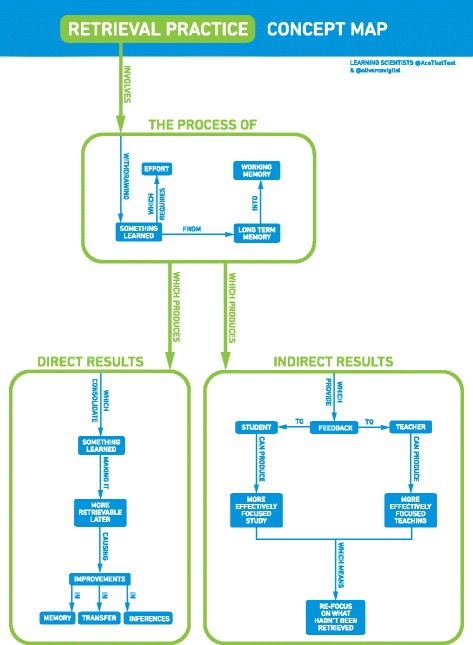
Concept map illustrating the process and resulting benefits of retrieval practice. Retrieval practice involves the process of withdrawing learned information from long-term memory into working memory, which requires effort. This produces direct benefits via the consolidation of learned information, making it easier to remember later and causing improvements in memory, transfer, and inferences. Retrieval practice also produces indirect benefits of feedback to students and teachers, which in turn can lead to more effective study and teaching practices, with a focus on information that was not accurately retrieved. *Copyright note:* this figure originally appeared in a blog post by the first and third authors (http://www.learningscientists.org/blog/2016/4/1-1)

**Fig. 4:**
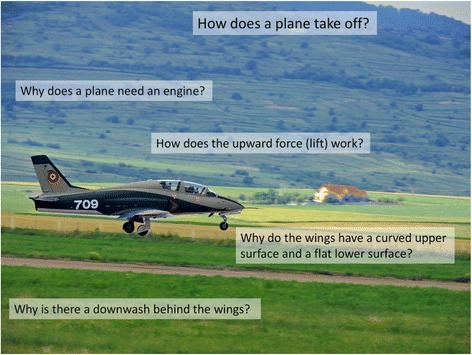
Illustration of “how” and “why” questions (i.e., elaborative interrogation questions) students might ask while studying the physics of flight. To help figure out how physics explains flight, students might ask themselves the following questions: “How does a plane take off?”; “Why does a plane need an engine?”; “How does the upward force (lift) work?”; “Why do the wings have a curved upper surface and a flat lower surface?”; and “Why is there a downwash behind the wings?”. *Copyright note:* the image of the plane was downloaded from Pixabay.com and is free to use, modify, and share

**Fig. 5:**
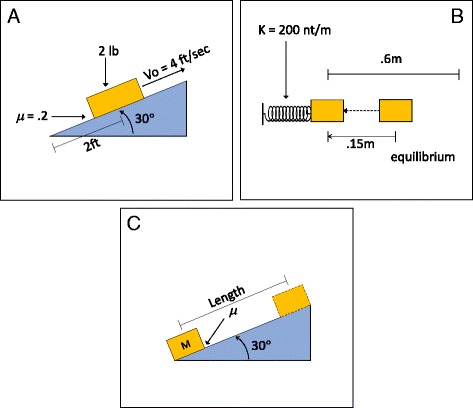
Three examples of physics problems that would be categorized differently by novices and experts. The problems in (**a**) and (**c**) look similar on the surface, so novices would group them together into one category. Experts, however, will recognize that the problems in (**b**) and (**c**) both relate to the principle of energy conservation, and so will group those two problems into one category instead. *Copyright note:* the figure was produced by the authors, based on figures in Chi et al. (1981)

**Fig. 6:**
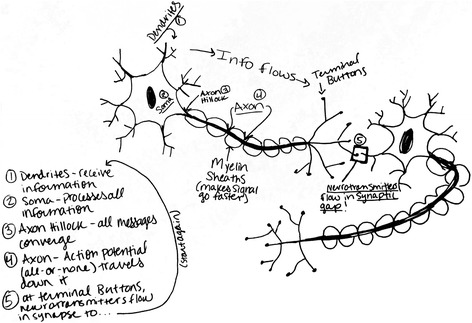
Example of how to enhance learning through use of a visual example. Students might view this visual representation of neural communications with the words provided, or they could draw a similar visual representation themselves. *Copyright note:* this figure was produced by the authors

**Fig. 7:**
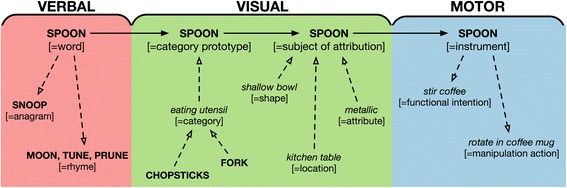
Example of word properties associated with visual, verbal, and motor coding for the word “SPOON”. A word can evoke multiple types of representation (“codes” in dual coding theory). Viewing a word will automatically evoke verbal representations related to its component letters and phonemes. Words representing objects (i.e., concrete nouns) will also evoke visual representations, including information about similar objects, component parts of the object, and information about where the object is typically found. In some cases, additional codes can also be evoked, such as motor-related properties of the represented object, where contextual information related to the object’s functional intention and manipulation action may also be processed automatically when reading the word. *Copyright note:* this figure was produced by the authors and is based on Aylwin (1990; Fig. 2) and Madan and Singhal (2012a, Fig. 3)

These informal writings, while allowing teachers to explore their approach to teaching practice (Luehmann, 2008), give us a unique window into the application of the science of learning to the classroom. By examining these blogs, we can not only observe how basic cognitive research is being applied in the classroom by teachers who are reading it, but also how it is being misapplied, and what questions teachers may be posing that have gone unaddressed in the scientific literature. Throughout this review, we illustrate each strategy with examples of how it can be implemented (see Table 1 and Figs. 1, 2, 3, 4, 5, 6 and 7), as well as with relevant teacher blog posts that reflect on its application, and draw upon this work to pin-point fruitful avenues for further basic and applied research.

**Table 1:** Six strategies for effective learning, each illustrated with an implementation example from the biological bases of behavior

Learning strategy	Description	Application examples (using biological bases of behavior from basic psychology)
Spaced practice	Creating a study schedule that spreads study activities out over time	Students can block off time to study and restudy key concepts such as action potentials and the nervous systems on multiple days before an exam, rather than repeatedly studying these concepts right before the exam
Interleaving	Switching between topics while studying	After studying the peripheral nervous system for a few minutes, students can switch to the sympathetic nervous system and then to the parasympathetic system; next time, students can study the three in a different order, noting what new connections they can make between them
Retrieval practice	Bringing learned information to mind from long-term memory	When learning about neural communication, students can practice writing out how neurons work together in the brain to send messages (from dendrites, to soma, to axon, to terminal buttons)
Elaboration	Asking and explaining why and how things work	Students can ask and explain why Botox prevents wrinkles: the nervous system cannot send messages to move certain muscles
Concrete examples	When studying abstract concepts, illustrating them with specific examples	Students can imagine the following example to explain the peripheral nervous system: a fire alarm goes off. The sympathetic nervous system allows people to move quickly out of the building; the parasympathetic system brings stress levels back down when the fire alarm turns off
Dual coding	Combining words with visuals	Students can draw two neurons and explain how one communicates with the other via the synaptic gap

## Spaced practice

The benefits of spaced (or distributed) practice to learning are arguably one of the strongest contributions that cognitive psychology has made to education (Kang, 2016). The effect is simple: the same amount of repeated studying of the same information spaced out over time will lead to greater retention of that information in the long run, compared with repeated studying of the same information for the same amount of time in one study session. The benefits of distributed practice were first empirically demonstrated in the 19^th^ century. As part of his extensive investigation into his own memory, Ebbinghaus (1885/1913) found that when he spaced out repetitions across 3 days, he could almost halve the number of repetitions necessary to relearn a series of 12 syllables in one day (Chapter 8). He thus concluded that “a suitable distribution of [repetitions] over a space of time is decidedly more advantageous than the massing of them at a single time” (Section 34). For those who want to read more about Ebbinghaus’s contribution to memory research, Roediger (1985) provides an excellent summary.

Since then, hundreds of studies have examined spacing effects both in the laboratory and in the classroom (Kang, 2016). Spaced practice appears to be particularly useful at large retention intervals: in the meta-analysis by Cepeda, Pashler, Vul, Wixted, and Rohrer (2006), all studies with a retention interval longer than a month showed a clear benefit of distributed practice. The “new theory of disuse” (Bjork & Bjork, 1992) provides a helpful mechanistic explanation for the benefits of spacing to learning. This theory posits that memories have both retrieval strength and storage strength. Whereas retrieval strength is thought to measure the ease with which a memory can be recalled at a given moment, storage strength (which cannot be measured directly) represents the extent to which a memory is truly embedded in the mind. When studying is taking place, both retrieval strength and storage strength receive a boost. However, the extent to which storage strength is boosted depends upon retrieval strength, and the relationship is negative: the greater the current retrieval strength, the smaller the gains in storage strength. Thus, the information learned through “cramming” will be rapidly forgotten due to high retrieval strength and low storage strength (Bjork & Bjork, 2011), whereas spacing out learning increases storage strength by allowing retrieval strength to wane before restudy.

Teachers can introduce spacing to their students in two broad ways. One involves creating opportunities to revisit information throughout the semester, or even in future semesters. This does involve some up-front planning, and can be difficult to achieve, given time constraints and the need to cover a set curriculum. However, spacing can be achieved with no great costs if teachers set aside a few minutes per class to review information from previous lessons. The second method involves putting the onus to space on the students themselves. Of course, this would work best with older students – high school and above. Because spacing requires advance planning, it is crucial that the teacher helps students plan their studying. For example, teachers could suggest that students schedule study sessions on days that alternate with the days on which a particular class meets (e.g., schedule review sessions for Tuesday and Thursday when the class meets Monday and Wednesday; see Fig. 1 for a more complete weekly spaced practice schedule). It important to note that the spacing effect refers to information that is repeated multiple times, rather than the idea of studying *different* material in one long session versus spaced out in small study sessions over time. However, for teachers and particularly for students planning a study schedule, the subtle difference between the two situations (spacing out restudy opportunities, versus spacing out studying of different information over time) may be lost. Future research should address the effects of spacing out studying of different information over time, whether the same considerations apply in this situation as compared to spacing out restudy opportunities, and how important it is for teachers and students to understand the difference between these two types of spaced practice.

It is important to note that students may feel less confident when they space their learning (Bjork, 1999) than when they cram. This is because spaced learning is harder – but it is this “desirable difficulty” that helps learning in the long term (Bjork, 1994). Students tend to cram for exams rather than space out their learning. One explanation for this is that cramming does “work”, if the goal is only to pass an exam. In order to change students’ minds about how they schedule their studying, it might be important to emphasize the value of retaining information beyond a final exam in one course.

Ideas for how to apply spaced practice in teaching have appeared in numerous teacher blogs (e.g., Fawcett, 2013; Kraft, 2015; Picciotto, 2009). In England in particular, as of 2013, high-school students need to be able to remember content from up to 3 years back on cumulative exams (General Certificate of Secondary Education (GCSE) and A-level exams; see CIFE, 2012). A-levels in particular determine what subject students study in university and which programs they are accepted into, and thus shape the path of their academic career. A common approach for dealing with these exams has been to include a “revision” (i.e., studying or cramming) period of a few weeks leading up to the high-stakes cumulative exams. Now, teachers who follow cognitive psychology are advocating a shift of priorities to spacing learning over time across the 3 years, rather than teaching a topic once and then intensely reviewing it weeks before the exam (Cox, 2016a; Wood, 2017). For example, some teachers have suggested using homework assignments as an opportunity for spaced practice by giving students homework on previous topics (Rose, 2014). However, questions remain, such as whether spaced practice can ever be effective enough to completely alleviate the need or utility of a cramming period (Cox, 2016b), and how one can possibly figure out the optimal lag for spacing (Benney, 2016; Firth, 2016).

There has been considerable research on the question of optimal lag, and much of it is quite complex; two sessions neither too close together (i.e., cramming) nor too far apart are ideal for retention. In a large-scale study, Cepeda, Vul, Rohrer, Wixted, and Pashler (2008) examined the effects of the gap between study sessions and the interval between study and test across long periods, and found that the optimal gap between study sessions was contingent on the retention interval. Thus, it is not clear how teachers can apply the complex findings on lag to their own classrooms.

A useful avenue of research would be to simplify the research paradigms that are used to study optimal lag, with the goal of creating a flexible, spaced-practice framework that teachers could apply and tailor to their own teaching needs. For example, an Excel macro spreadsheet was recently produced to help teachers plan for lagged lessons (Weinstein-Jones & Weinstein, 2017; see Weinstein & Weinstein-Jones (2017) for a description of the algorithm used in the spreadsheet), and has been used by teachers to plan their lessons (Penfound, 2017). However, one teacher who found this tool helpful also wondered whether the more sophisticated plan was any better than his own method of manually selecting poorly understood material from previous classes for later review (Lovell, 2017). This direction is being actively explored within personalized online learning environments (Kornell & Finn, 2016; Lindsey, Shroyer, Pashler, & Mozer, 2014), but teachers in physical classrooms might need less technologically-driven solutions to teach cohorts of students.

It seems teachers would greatly appreciate a set of guidelines for how to implement spacing in the curriculum in the most effective, but also the most efficient manner. While the cognitive field has made great advances in terms of understanding the mechanisms behind spacing, what teachers need more of are concrete evidence-based tools and guidelines for direct implementation in the classroom. These could include more sophisticated and experimentally tested versions of the software described above (Weinstein-Jones & Weinstein, 2017), or adaptable templates of spaced curricula. Moreover, researchers need to evaluate the effectiveness of these tools in a real classroom environment, over a semester or academic year, in order to give pedagogically relevant evidence-based recommendations to teachers.

## Interleaving

Another scheduling technique that has been shown to increase learning is interleaving. Interleaving occurs when different ideas or problem types are tackled in a sequence, as opposed to the more common method of attempting multiple versions of the same problem in a given study session (known as blocking). Interleaving as a principle can be applied in many different ways. One such way involves interleaving different types of problems during learning, which is particularly applicable to subjects such as math and physics (see Fig. 2a for an example with fractions, based on a study by Patel, Liu, & Koedinger, 2016). For example, in a study with college students, Rohrer and Taylor (2007) found that shuffling math problems that involved calculating the volume of different shapes resulted in better test performance 1 week later than when students answered multiple problems about the same type of shape in a row. This pattern of results has also been replicated with younger students, for example 7^th^ grade students learning to solve graph and slope problems (Rohrer, Dedrick, & Stershic, 2015). The proposed explanation for the benefit of interleaving is that switching between different problem types allows students to acquire the ability to choose the right method for solving different types of problems rather than learning only the method itself, and not when to apply it.

Do the benefits of interleaving extend beyond problem solving? The answer appears to be yes. Interleaving can be helpful in other situations that require discrimination, such as inductive learning. Kornell and Bjork (2008) examined the effects of interleaving in a task that might be pertinent to a student of the history of art: the ability to match paintings to their respective painters. Students who studied different painters’ paintings interleaved at study were more successful on a later identification test than were participants who studied the paintings blocked by painter. Birnbaum, Kornell, Bjork, and Bjork (2013) proposed the discriminative-contrast hypothesis to explain that interleaving enhances learning by allowing the comparison between exemplars of different categories. They found support for this hypothesis in a set of experiments with bird categorization: participants benefited from interleaving and also from spacing, but not when the spacing interrupted side-by-side comparisons of birds from different categories.

Another type of interleaving involves the interleaving of study and test opportunities. This type of interleaving has been applied, once again, to problem solving, whereby students alternate between attempting a problem and viewing a worked example (Trafton & Reiser, 1993); this pattern appears to be superior to answering a string of problems in a row, at least with respect to the amount of time it takes to achieve mastery of a procedure (Corbett, Reed, Hoffmann, MacLaren, & Wagner, 2010). The benefits of interleaving study and test opportunities – rather than blocking study followed by attempting to answer problems or questions – might arise due to a process known as “test-potentiated learning”. That is, a study opportunity that immediately follows a retrieval attempt may be more fruitful than when that same studying was not preceded by retrieval (Arnold & McDermott, 2013).

For problem-based subjects, the interleaving technique is straightforward: simply mix questions on homework and quizzes with previous materials (which takes care of spacing as well); for languages, mix vocabulary themes rather than blocking by theme (Thomson & Mehring, 2016). But interleaving as an educational strategy ought to be presented to teachers with some caveats. Research has focused on interleaving material that is somewhat related (e.g., solving different mathematical equations, Rohrer et al., 2015), whereas students sometimes ask whether they should interleave material from different subjects – a practice that has not received empirical support (Hausman & Kornell, 2014). When advising students how to study independently, teachers should thus proceed with caution. Since it is easy for younger students to confuse this type of unhelpful interleaving with the more helpful interleaving of related information, it may be best for teachers of younger grades to create opportunities for interleaving in homework and quiz assignments rather than putting the onus on the students themselves to make use of the technique. Technology can be very helpful here, with apps such as Quizlet, Memrise, Anki, Synap, Quiz Champ, and many others (see also “Learning Scientists”, 2017) that not only allow instructor-created quizzes to be taken by students, but also provide built-in interleaving algorithms so that the burden does not fall on the teacher or the student to carefully plan which items are interleaved when.

An important point to consider is that in educational practice, the distinction between spacing and interleaving can be difficult to delineate. The gap between the scientific and classroom definitions of interleaving is demonstrated by teachers’ own writings about this technique. When they write about interleaving, teachers often extend the term to connote a curriculum that involves returning to topics multiple times throughout the year (e.g., Kirby, 2014; see “Learning Scientists” (2016a) for a collection of similar blog posts by several other teachers). The “interleaving” of topics throughout the curriculum produces an effect that is more akin to what cognitive psychologists call “spacing” (see Fig. 2b for a visual representation of the difference between interleaving and spacing). However, cognitive psychologists have not examined the effects of structuring the curriculum in this way, and open questions remain: does repeatedly circling back to previous topics throughout the semester interrupt the learning of new information? What are some effective techniques for interleaving old and new information within one class? And how does one determine the balance between old and new information?

## Retrieval practice

While tests are most often used in educational settings for assessment, a lesser-known benefit of tests is that they actually improve memory of the tested information. If we think of our memories as libraries of information, then it may seem surprising that retrieval (which happens when we take a test) improves memory; however, we know from a century of research that retrieving knowledge actually strengthens it (see Karpicke, Lehman, & Aue, 2014). Testing was shown to strengthen memory as early as 100 years ago (Gates, 1917), and there has been a surge of research in the last decade on the mnemonic benefits of testing, or *retrieval practice*. Most of the research on the effectiveness of retrieval practice has been done with college students (see Roediger & Karpicke, 2006; Roediger, Putnam, & Smith, 2011), but retrieval-based learning has been shown to be effective at producing learning for a wide range of ages, including preschoolers (Fritz, Morris, Nolan, & Singleton, 2007), elementary-aged children (e.g., Karpicke, Blunt, & Smith, 2016; Karpicke, Blunt, Smith, & Karpicke, 2014; Lipko-Speed, Dunlosky, & Rawson, 2014; Marsh, Fazio, & Goswick, 2012; Ritchie, Della Sala, & McIntosh, 2013), middle-school students (e.g., McDaniel, Thomas, Agarwal, McDermott, & Roediger, 2013; McDermott, Agarwal, D’Antonio, Roediger, & McDaniel, 2014), and high-school students (e.g., McDermott et al., 2014). In addition, the effectiveness of retrieval-based learning has been extended beyond simple testing to other activities in which retrieval practice can be integrated, such as concept mapping (Blunt & Karpicke, 2014; Karpicke, Blunt, et al., 2014; Ritchie et al., 2013).

A debate is currently ongoing as to the effectiveness of retrieval practice for more complex materials (Karpicke & Aue, 2015; Roelle & Berthold, 2017; Van Gog & Sweller, 2015). Practicing retrieval has been shown to improve the application of knowledge to new situations (e.g., Butler, 2010; Dirkx, Kester, & Kirschner, 2014); McDaniel et al., 2013; Smith, Blunt, Whiffen, & Karpicke, 2016); but see Tran, Rohrer, and Pashler (2015) and Wooldridge, Bugg, McDaniel, and Liu (2014), for retrieval practice studies that showed limited or no increased transfer compared to restudy. Retrieval practice effects on higher-order learning may be more sensitive than fact learning to encoding factors, such as the way material is presented during study (Eglington & Kang, 2016). In addition, retrieval practice may be more beneficial for higher-order learning if it includes more scaffolding (Fiechter & Benjamin, 2017; but see Smith, Blunt, et al., 2016) and targeted practice with application questions (Son & Rivas, 2016).

How does retrieval practice help memory? Figure 3 illustrates both the direct and indirect benefits of retrieval practice identified by the literature. The act of retrieval itself is thought to strengthen memory (Karpicke, Blunt, et al., 2014; Roediger & Karpicke, 2006; Smith, Roediger, & Karpicke, 2013). For example, Smith et al. (2013) showed that if students brought information to mind without actually producing it (covert retrieval), they remembered the information just as well as if they overtly produced the retrieved information (overt retrieval). Importantly, both overt and covert retrieval practice improved memory over control groups without retrieval practice, even when feedback was not provided. The fact that bringing information to mind in the absence of feedback or restudy opportunities improves memory leads researchers to conclude that it is the act of retrieval – thinking back to bring information to mind – that improves memory of that information.

The benefit of retrieval practice depends to a certain extent on successful retrieval (see Karpicke, Lehman, et al., 2014). For example, in Experiment 4 of Smith et al. (2013), students successfully retrieved 72% of the information during retrieval practice. Of course, retrieving 72% of the information was compared to a restudy control group, during which students were re-exposed to 100% of the information, creating a bias in favor of the restudy condition. Yet retrieval led to superior memory later compared to the restudy control. However, if retrieval success is extremely low, then it is unlikely to improve memory (e.g., Karpicke, Blunt, et al., 2014), particularly in the absence of feedback. On the other hand, if retrieval-based learning situations are constructed in such a way that ensures high levels of success, the act of bringing the information to mind may be undermined, thus making it less beneficial. For example, if a student reads a sentence and then immediately covers the sentence and recites it out loud, they are likely not retrieving the information but rather just keeping the information in their working memory long enough to recite it again (see Smith, Blunt, et al., 2016 for a discussion of this point). Thus, it is important to balance success of retrieval with overall difficulty in retrieving the information (Smith & Karpicke, 2014; Weinstein, Nunes, & Karpicke, 2016). If initial retrieval success is low, then feedback can help improve the overall benefit of practicing retrieval (Kang, McDermott, & Roediger, 2007; Smith & Karpicke, 2014). Kornell, Klein, and Rawson (2015), however, found that it was the retrieval attempt and not the correct production of information that produced the retrieval practice benefit – as long as the correct answer was provided after an unsuccessful attempt, the benefit was the same as for a successful retrieval attempt in this set of studies. From a practical perspective, it would be helpful for teachers to know when retrieval attempts in the absence of success are helpful, and when they are not. There may also be additional reasons beyond retrieval benefits that would push teachers towards retrieval practice activities that produce some success amongst students; for example, teachers may hesitate to give students retrieval practice exercises that are too difficult, as this may negatively affect self-efficacy and confidence.

In addition to the fact that bringing information to mind directly improves memory for that information, engaging in retrieval practice can produce indirect benefits as well (see Roediger et al., 2011). For example, research by Weinstein, Gilmore, Szpunar, and McDermott (2014) demonstrated that when students expected to be tested, the increased test expectancy led to better-quality encoding of new information. Frequent testing can also serve to decrease mind-wandering – that is, thoughts that are unrelated to the material that students are supposed to be studying (Szpunar, Khan, & Schacter, 2013).

Practicing retrieval is a powerful way to improve meaningful learning of information, and it is relatively easy to implement in the classroom. For example, requiring students to practice retrieval can be as simple as asking students to put their class materials away and try to write out everything they know about a topic. Retrieval-based learning strategies are also flexible. Instructors can give students practice tests (e.g., short-answer or multiple-choice, see Smith & Karpicke, 2014), provide open-ended prompts for the students to recall information (e.g., Smith, Blunt, et al., 2016) or ask their students to create concept maps from memory (e.g., Blunt & Karpicke, 2014). In one study, Weinstein et al. (2016) looked at the effectiveness of inserting simple short-answer questions into online learning modules to see whether they improved student performance. Weinstein and colleagues also manipulated the placement of the questions. For some students, the questions were interspersed throughout the module, and for other students the questions were all presented at the end of the module. Initial success on the short-answer questions was higher when the questions were interspersed throughout the module. However, on a later test of learning from that module, the original placement of the questions in the module did not matter for performance. As with spaced practice, where the optimal gap between study sessions is contingent on the retention interval, the optimum difficulty and level of success during retrieval practice may also depend on the retention interval. Both groups of students who answered questions performed better on the delayed test compared to a control group without question opportunities during the module. Thus, the important thing is for instructors to provide opportunities for retrieval practice during learning. Based on previous research, any activity that promotes the successful retrieval of information should improve learning.

Retrieval practice has received a lot of attention in teacher blogs (see “Learning Scientists” (2016b) for a collection). A common theme seems to be an emphasis on low-stakes (Young, 2016) and even no-stakes (Cox, 2015) testing, the goal of which is to increase learning rather than assess performance. In fact, one well-known charter school in the UK has an official homework policy grounded in retrieval practice: students are to test themselves on subject knowledge for 30 minutes every day in lieu of standard homework (Michaela Community School, 2014). The utility of homework, particularly for younger children, is often a hotly debated topic outside of academia (e.g., Shumaker, 2016; but see Jones (2016) for an opposing viewpoint and Cooper (1989) for the original research the blog posts were based on). Whereas some research shows clear links between homework and academic achievement (Valle et al., 2016), other researchers have questioned the effectiveness of homework (Dettmers, Trautwein, & Lüdtke, 2009). Perhaps amending homework to involve retrieval practice might make it more effective; this remains an open empirical question.

One final consideration is that of test anxiety. While retrieval practice can be very powerful at improving memory, some research shows that pressure during retrieval can undermine some of the learning benefit. For example, Hinze and Rapp (2014) manipulated pressure during quizzing to create high-pressure and low-pressure conditions. On the quizzes themselves, students performed equally well. However, those in the high-pressure condition did not perform as well on a criterion test later compared to the low-pressure group. Thus, test anxiety may reduce the learning benefit of retrieval practice. Eliminating all high-pressure tests is probably not possible, but instructors can provide a number of low-stakes retrieval opportunities for students to help increase learning. The use of low-stakes testing can serve to decrease test anxiety (Khanna, 2015), and has recently been shown to negate the detrimental impact of stress on learning (Smith, Floerke, & Thomas, 2016). This is a particularly important line of inquiry to pursue for future research, because many teachers who are not familiar with the effectiveness of retrieval practice may be put off by the implied pressure of “testing”, which evokes the much maligned high-stakes standardized tests (e.g., McHugh, 2013).

## Elaboration

Elaboration involves connecting new information to pre-existing knowledge. Anderson (1983, p.285) made the following claim about elaboration: “One of the most potent manipulations that can be performed in terms of increasing a subject’s memory for material is to have the subject elaborate on the to-be-remembered material.” Postman (1976, p. 28) defined elaboration most parsimoniously as “additions to nominal input”, and Hirshman (2001, p. 4369) provided an elaboration on this definition (pun intended!), defining elaboration as “A conscious, intentional process that associates to-be-remembered information with other information in memory.” However, in practice, elaboration could mean many different things. The common thread in all the definitions is that elaboration involves adding features to an existing memory.

One possible instantiation of elaboration is thinking about information on a deeper level. The levels (or “depth”) of processing framework, proposed by Craik and Lockhart (1972), predicts that information will be remembered better if it is processed more deeply in terms of meaning, rather than shallowly in terms of form. The leves of processing framework has, however, received a number of criticisms (Craik, 2002). One major problem with this framework is that it is difficult to measure “depth”. And if we are not able to actually measure depth, then the argument can become circular: is it that something was remembered better because it was studied more deeply, or do we conclude that it must have been studied more deeply because it is remembered better? (See Lockhart & Craik, 1990, for further discussion of this issue).

Another mechanism by which elaboration can confer a benefit to learning is via improvement in organization (Bellezza, Cheesman, & Reddy, 1977; Mandler, 1979). By this view, elaboration involves making information more integrated and organized with existing knowledge structures. By connecting and integrating the to-be-learned information with other concepts in memory, students can increase the extent to which the ideas are organized in their minds, and this increased organization presumably facilitates the reconstruction of the past at the time of retrieval.

Elaboration is such a broad term and can include so many different techniques that it is hard to claim that elaboration will always help learning. There is, however, a specific technique under the umbrella of elaboration for which there is relatively strong evidence in terms of effectiveness (Dunlosky et al., 2013; Pashler et al., 2007). This technique is called elaborative interrogation, and involves students questioning the materials that they are studying (Pressley, McDaniel, Turnure, Wood, & Ahmad, 1987). More specifically, students using this technique would ask “how” and “why” questions about the concepts they are studying (see Fig. 4 for an example on the physics of flight). Then, crucially, students would try to answer these questions – either from their materials or, eventually, from memory (McDaniel & Donnelly, 1996). The process of figuring out the answer to the questions – with some amount of uncertainty (Overoye & Storm, 2015) – can help learning. When using this technique, however, it is important that students check their answers with their materials or with the teacher; when the content generated through elaborative interrogation is poor, it can actually hurt learning (Clinton, Alibali, & Nathan, 2016).

Students can also be encouraged to self-explain concepts to themselves while learning (Chi, De Leeuw, Chiu, & LaVancher, 1994). This might involve students simply saying out loud what steps they need to perform to solve an equation. Aleven and Koedinger (2002) conducted two classroom studies in which students were either prompted by a “cognitive tutor” to provide self-explanations during a problem-solving task or not, and found that the self-explanations led to improved performance. According to the authors, this approach could scale well to real classrooms. If possible and relevant, students could even perform actions alongside their self-explanations (Cohen, 1981; see also the enactment effect, Hainselin, Picard, Manolli, Vankerkore-Candas, & Bourdin, 2017). Instructors can scaffold students in these types of activities by providing self-explanation prompts throughout to-be-learned material (O’Neil et al., 2014). Ultimately, the greatest potential benefit of accurate self-explanation or elaboration is that the student will be able to transfer their knowledge to a new situation (Rittle-Johnson, 2006).

The technical term “elaborative interrogation” has not made it into the vernacular of educational bloggers (a search on https://educationechochamberuncut.wordpress.com, which consolidates over 3,000 UK-based teacher blogs, yielded zero results for that term). However, a few teachers have blogged about elaboration more generally (e.g., Hobbiss, 2016) and deep questioning specifically (e.g., Class Teaching, 2013), just without using the specific terminology. This strategy in particular may benefit from a more open dialog between researchers and teachers to facilitate the use of elaborative interrogation in the classroom and to address possible barriers to implementation. In terms of advancing the scientific understanding of elaborative interrogation in a classroom setting, it would be informative to conduct a larger-scale intervention to see whether having students elaborate during reading actually helps their understanding. It would also be useful to know whether the students really need to generate their own elaborative interrogation (“how” and “why”) questions, versus answering questions provided by others. How long should students persist to find the answers? When is the right time to have students engage in this task, given the levels of expertise required to do it well (Clinton et al., 2016)? Without knowing the answers to these questions, it may be too early for us to instruct teachers to use this technique in their classes. Finally, elaborative interrogation takes a long time. Is this time efficiently spent? Or, would it be better to have the students try to answer a few questions, pool their information as a class, and then move to practicing retrieval of the information?

## Concrete examples

Providing supporting information can improve the learning of key ideas and concepts. Specifically, using concrete examples to supplement content that is more conceptual in nature can make the ideas easier to understand and remember. Concrete examples can provide several advantages to the learning process: (a) they can concisely convey information, (b) they can provide students with more concrete information that is easier to remember, and (c) they can take advantage of the superior memorability of pictures relative to words (see “Dual Coding”).

Words that are more concrete are both recognized and recalled better than abstract words (Gorman, 1961; e.g., “button” and “bound,” respectively). Furthermore, it has been demonstrated that information that is more concrete and imageable enhances the learning of associations, even with abstract content (Caplan & Madan, 2016; Madan, Glaholt, & Caplan, 2010; Paivio, 1971). Following from this, providing concrete examples during instruction should improve retention of related abstract concepts, rather than the concrete examples alone being remembered better. Concrete examples can be useful both during instruction and during practice problems. Having students actively explain how two examples are similar and encouraging them to extract the underlying structure on their own can also help with transfer. In a laboratory study, Berry (1983) demonstrated that students performed well when given concrete practice problems, regardless of the use of verbalization (akin to elaborative interrogation), but that verbalization helped students transfer understanding from concrete to abstract problems. One particularly important area of future research is determining how students can best make the link between concrete examples and abstract ideas.

Since abstract concepts are harder to grasp than concrete information (Paivio, Walsh, & Bons, 1994), it follows that teachers ought to illustrate abstract ideas with concrete examples. However, care must be taken when selecting the examples. LeFevre and Dixon (1986) provided students with both concrete examples and abstract instructions and found that when these were inconsistent, students followed the concrete examples rather than the abstract instructions, potentially constraining the application of the abstract concept being taught. Lew, Fukawa-Connelly, Mejí-Ramos, and Weber (2016) used an interview approach to examine why students may have difficulty understanding a lecture. Responses indicated that some issues were related to understanding the overarching topic rather than the component parts, and to the use of informal colloquialisms that did not clearly follow from the material being taught. Both of these issues could have potentially been addressed through the inclusion of a greater number of relevant concrete examples.

One concern with using concrete examples is that students might only remember the examples – especially if they are particularly memorable, such as fun or gimmicky examples – and will not be able to transfer their understanding from one example to another, or more broadly to the abstract concept. However, there does not seem to be any evidence that fun relevant examples actually hurt learning by harming memory for important information. Instead, fun examples and jokes tend to be more memorable, but this boost in memory for the joke does not seem to come at a cost to memory for the underlying concept (Baldassari & Kelley, 2012). However, two important caveats need to be highlighted. First, to the extent that the more memorable content is not relevant to the concepts of interest, learning of the target information can be compromised (Harp & Mayer, 1998). Thus, care must be taken to ensure that all examples and gimmicks are, in fact, related to the core concepts that the students need to acquire, and do not contain irrelevant perceptual features (Kaminski & Sloutsky, 2013).

The second issue is that novices often notice and remember the surface details of an example rather than the underlying structure. Experts, on the other hand, can extract the underlying structure from examples that have divergent surface features (Chi, Feltovich, & Glaser, 1981; see Fig. 5 for an example from physics). Gick and Holyoak (1983) tried to get students to apply a rule from one problem to another problem that appeared different on the surface, but was structurally similar. They found that providing multiple examples helped with this transfer process compared to only using one example – especially when the examples provided had different surface details. More work is also needed to determine how many examples are sufficient for generalization to occur (and this, of course, will vary with contextual factors and individual differences). Further research on the continuum between concrete/specific examples and more abstract concepts would also be informative. That is, if an example is not concrete enough, it may be too difficult to understand. On the other hand, if the example is too concrete, that could be detrimental to generalization to the more abstract concept (although a diverse set of very concrete examples may be able to help with this). In fact, in a controversial article, Kaminski, Sloutsky, and Heckler (2008) claimed that abstract examples were more effective than concrete examples. Later rebuttals of this paper contested whether the abstract versus concrete distinction was clearly defined in the original study (see Reed, 2008, for a collection of letters on the subject). This ideal point along the concrete-abstract continuum might also interact with development.

Finding teacher blog posts on concrete examples proved to be more difficult than for the other strategies in this review. One optimistic possibility is that teachers frequently use concrete examples in their teaching, and thus do not think of this as a specific contribution from cognitive psychology; the one blog post we were able to find that discussed concrete examples suggests that this might be the case (Boulton, 2016). The idea of “linking abstract concepts with concrete examples” is also covered in 25% of teacher-training textbooks used in the US, according to the report by Pomerance et al. (2016); this is the second most frequently covered of the six strategies, after “posing probing questions” (i.e., elaborative interrogation). A useful direction for future research would be to establish how teachers are using concrete examples in their practice, and whether we can make any suggestions for improvement based on research into the science of learning. For example, if two examples are better than one (Bauernschmidt, 2017), are additional examples also needed, or are there diminishing returns from providing more examples? And, how can teachers best ensure that concrete examples are consistent with prior knowledge (Reed, 2008)?

## Dual coding

Both the memory literature and folk psychology support the notion of visual examples being beneficial—the adage of “a picture is worth a thousand words” (traced back to an advertising slogan from the 1920s; Meider, 1990). Indeed, it is well-understood that more information can be conveyed through a simple illustration than through several paragraphs of text (e.g., Barker & Manji, 1989; Mayer & Gallini, 1990). Illustrations can be particularly helpful when the described concept involves several parts or steps and is intended for individuals with low prior knowledge (Eitel & Scheiter, 2015; Mayer & Gallini, 1990). Figure 6 provides a concrete example of this, illustrating how information can flow through neurons and synapses.

In addition to being able to convey information more succinctly, pictures are also more memorable than words (Paivio & Csapo, 1969, 1973). In the memory literature, this is referred to as the *picture superiority effect*, and dual coding theory was developed in part to explain this effect. Dual coding follows from the notion of text being accompanied by complementary visual information to enhance learning. Paivio (1971, 1986) proposed dual coding theory as a mechanistic account for the integration of multiple information “codes” to process information. In this theory, a code corresponds to a modal or otherwise distinct representation of a concept—e.g., “mental images for ‘book’ have visual, tactual, and other perceptual qualities similar to those evoked by the referent objects on which the images are based” (Clark & Paivio, 1991, p. 152). Aylwin (1990) provides a clear example of how the word “dog” can evoke verbal, visual, and enactive representations (see Fig. 7 for a similar example for the word “SPOON”, based on Aylwin, 1990 (Fig. 2) and Madan & Singhal, 2012a (Fig. 3)). Codes can also correspond to emotional properties (Clark & Paivio, 1991; Paivio, 2013). Clark and Paivio (1991) provide a thorough review of dual coding theory and its relation to education, while Paivio (2007) provides a comprehensive treatise on dual coding theory. Broadly, dual coding theory suggests that providing multiple representations of the same information enhances learning and memory, and that information that more readily evokes additional representations (through automatic imagery processes) receives a similar benefit.

Paivio and Csapo (1973) suggest that verbal and imaginal codes have independent and additive effects on memory recall. Using visuals to improve learning and memory has been particularly applied to vocabulary learning (Danan, 1992; Sadoski, 2005), but has also shown success in other domains such as in health care (Hartland, Biddle, & Fallacaro, 2008). To take advantage of dual coding, verbal information should be accompanied by a visual representation when possible. However, while the studies discussed all indicate that the use of multiple representations of information is favorable, it is important to acknowledge that each representation also increases cognitive load and can lead to over-saturation (Mayer & Moreno, 2003).

Given that pictures are generally remembered better than words, it is important to ensure that the pictures students are provided with are helpful and relevant to the content they are expected to learn. McNeill, Uttal, Jarvin, and Sternberg (2009) found that providing visual examples decreased conceptual errors. However, McNeill et al. also found that when students were given visually rich examples, they performed more poorly than students who were not given any visual example, suggesting that the visual details can at times become a distraction and hinder performance. Thus, it is important to consider that images used in teaching are clear and not ambiguous in their meaning (Schwartz, 2007).

Further broadening the scope of dual coding theory, Engelkamp and Zimmer (1984) suggest that motor movements, such as “turning the handle,” can provide an additional motor code that can improve memory, linking studies of motor actions (enactment) with dual coding theory (Clark & Paivio, 1991; Engelkamp & Cohen, 1991; Madan & Singhal, 2012c). Indeed, enactment effects appear to primarily occur during learning, rather than during retrieval (Peterson & Mulligan, 2010). Along similar lines, Wammes, Meade, and Fernandes (2016) demonstrated that generating drawings can provide memory benefits beyond what could otherwise be explained by visual imagery, picture superiority, and other memory enhancing effects. Providing convergent evidence, even when overt motor actions are not critical in themselves, words representing functional objects have been shown to enhance later memory (Madan & Singhal, 2012b; Montefinese, Ambrosini, Fairfield, & Mammarella, 2013). This indicates that motoric processes can improve memory similarly to visual imagery, similar to memory differences for concrete vs. abstract words. Further research suggests that automatic motor simulation for functional objects is likely responsible for this memory benefit (Madan, Chen, & Singhal, 2016).

When teachers combine visuals and words in their educational practice, however, they may not always be taking advantage of dual coding – at least, not in the optimal manner. For example, a recent discussion on Twitter centered around one teacher’s decision to have 7^th^ Grade students replace certain words in their science laboratory report with a picture of that word (e.g., the instructions read “using a syringe …” and a picture of a syringe replaced the word; Turner, 2016a). Other teachers argued that this was not dual coding (Beaven, 2016; Williams, 2016), because there were no longer two different representations of the information. The first teacher maintained that dual coding was preserved, because this laboratory report with pictures was to be used alongside the original, fully verbal report (Turner, 2016b). This particular implementation – having students replace individual words with pictures – has not been examined in the cognitive literature, presumably because no benefit would be expected. In any case, we need to be clearer about implementations for dual coding, and more research is needed to clarify how teachers can make use of the benefits conferred by multiple representations and picture superiority.

Critically, dual coding theory is distinct from the notion of “learning styles,” which describe the idea that individuals benefit from instruction that matches their modality preference. While this idea is pervasive and individuals often subjectively feel that they have a preference, evidence indicates that the learning styles theory is not supported by empirical findings (e.g., Kavale, Hirshoren, & Forness, 1998; Pashler, McDaniel, Rohrer, & Bjork, 2008; Rohrer & Pashler, 2012). That is, there is no evidence that instructing students in their preferred learning style leads to an overall improvement in learning (the “meshing” hypothesis). Moreover, learning styles have come to be described as a myth or urban legend within psychology (Coffield, Moseley, Hall, & Ecclestone, 2004; Hattie & Yates, 2014; Kirschner & van Merriënboer, 2013; Kirschner, 2017); skepticism about learning styles is a common stance amongst evidence-informed teachers (e.g., Saunders, 2016). Providing evidence against the notion of learning styles, Kraemer, Rosenberg, and Thompson-Schill (2009) found that individuals who scored as “verbalizers” and “visualizers” did not perform any better on experimental trials matching their preference. Instead, it has recently been shown that learning through one’s preferred learning style is associated with elevated subjective judgements of learning, but not objective performance (Knoll, Otani, Skeel, & Van Horn, 2017). In contrast to learning styles, dual coding is based on providing additional, complementary forms of information to enhance learning, rather than tailoring instruction to individuals’ preferences.

## Conclusion

Genuine educational environments present many opportunities for combining the strategies outlined above. Spacing can be particularly potent for learning if it is combined with retrieval practice. The additive benefits of retrieval practice and spacing can be gained by engaging in retrieval practice multiple times (also known as distributed practice; see Cepeda et al., 2006). Interleaving naturally entails spacing if students interleave old and new material. Concrete examples can be both verbal and visual, making use of dual coding. In addition, the strategies of elaboration, concrete examples, and dual coding all work best when used as part of retrieval practice. For example, in the concept-mapping studies mentioned above (Blunt & Karpicke, 2014; Karpicke, Blunt, et al., 2014), creating concept maps while looking at course materials (e.g., a textbook) was not as effective for later memory as creating concept maps from memory. When practicing elaborative interrogation, students can start off answering the “how” and “why” questions they pose for themselves using class materials, and work their way up to answering them from memory. And when interleaving different problem types, students should be practicing answering them rather than just looking over worked examples.

But while these ideas for strategy combinations have empirical bases, it has not yet been established whether the benefits of the strategies to learning are additive, super-additive, or, in some cases, incompatible. Thus, future research needs to (a) better formalize the definition of each strategy (particularly critical for elaboration and dual coding), (b) identify best practices for implementation in the classroom, (c) delineate the boundary conditions of each strategy, and (d) strategically investigate interactions between the six strategies we outlined in this manuscript.
